# An evidence map of the effect of Tai Chi on health outcomes

**DOI:** 10.1186/s13643-016-0300-y

**Published:** 2016-07-27

**Authors:** Michele R. Solloway, Stephanie L. Taylor, Paul G. Shekelle, Isomi M. Miake-Lye, Jessica M. Beroes, Roberta M. Shanman, Susanne Hempel

**Affiliations:** 1 VA Greater Los Angeles Healthcare System, Los Angeles, CA USA; 2 VA Evidence-based Synthesis Program (ESP) Center, Los Angeles, CA USA; 3 University of California, Los Angeles, CA USA; 4 Evidence-based Practice Center (EPC), RAND Corporation, Santa Monica, CA USA

**Keywords:** Systematic review, Tai Chi, Evidence map, Health

## Abstract

**Background:**

This evidence map describes the volume and focus of Tai Chi research reporting health outcomes. Originally developed as a martial art, Tai Chi is typically taught as a series of slow, low-impact movements that integrate the breath, mind, and physical activity to achieve greater awareness and a sense of well-being.

**Methods:**

The evidence map is based on a systematic review of systematic reviews. We searched 11 electronic databases from inception to February 2014, screened reviews of reviews, and consulted with topic experts. We used a bubble plot to graphically display clinical topics, literature size, number of reviews, and a broad estimate of effectiveness.

**Results:**

The map is based on 107 systematic reviews. Two thirds of the reviews were published in the last five years. The topics with the largest number of published randomized controlled trials (RCTs) were general health benefits (51 RCTs), psychological well-being (37 RCTs), interventions for older adults (31 RCTs), balance (27 RCTs), hypertension (18 RCTs), fall prevention (15 RCTs), and cognitive performance (11 RCTs). The map identified a number of areas with evidence of a potentially positive treatment effect on patient outcomes, including Tai Chi for hypertension, fall prevention outside of institutions, cognitive performance, osteoarthritis, depression, chronic obstructive pulmonary disease, pain, balance confidence, and muscle strength. However, identified reviews cautioned that firm conclusions cannot be drawn due to methodological limitations in the original studies and/or an insufficient number of existing research studies.

**Conclusions:**

Tai Chi has been applied in diverse clinical areas, and for a number of these, systematic reviews have indicated promising results. The evidence map provides a visual overview of Tai Chi research volume and content.

**Systematic review registration:**

PROSPERO CRD42014009907

**Electronic supplementary material:**

The online version of this article (doi:10.1186/s13643-016-0300-y) contains supplementary material, which is available to authorized users.

## Background

Tai Chi, also known as T’ai chi ch’uan or Taijiquan, developed as an ancient Chinese martial art and is today widely practiced for its health benefits. Many forms of Tai Chi exist, but in western culture, it is most commonly taught as a series of slow, gentle, low-impact movements that integrate the breath, mind, and physical activity to achieve greater awareness and a sense of inner peace and well-being. The meditative movement is designed to strengthen and stretch the body, improve the flow of blood and other fluids, improve balance, proprioception, and awareness of how the body moves through space; and it may be practiced in a group format or alone [1]. Results from the 2007 National Health Interview Survey—a survey of a representative sample of adults in the USA—estimated that approximately 2.3 million adults in the USA practiced Tai Chi in the past 12 months. There is no official licensure granted by national or state professional boards, and there are no official standards for training instructors; thus, individual training programs vary.

Research on effects of Tai Chi on health outcomes continues to expand and has been the subject of many primary research studies and reviews of the literature. The research field covers a wide spectrum of clinical indications, targets a range of populations, and has focused on a variety of settings. A systematic review of systematic reviews identified 35 reviews published in 2010 and concluded that Tai Chi is effective for fall prevention and improving psychological health and was associated with general health benefits for older people [2]. However, the interest in Tai Chi has increased in particular in recent years and since 2010, more than twice as many systematic reviews have been published. In order to provide a broad overview of the research evidence that has been published to date, we conducted a systematic review of systematic reviews of the effects of Tai Chi on health outcomes [3].

We present the results of the systematic review of systematic reviews as an evidence map, a form of systematic literature synthesis that uses visual displays of the volume and content areas of research. Evidence maps are an emerging evidence synthesis tool that aim to provide an overview over large research areas [4]. The evidence map presents a summary of the focus of Tai Chi research that contributes to the evidence base on patient health outcomes in a format that is easily accessible to healthcare practitioners and policy makers and other stakeholders. The objective of the evidence map is to indicate the research focus and show the presence as well as the absence of published research for individual topic areas; the evidence map may inform research agendas or be used as a signpost for practitioners.

## Methods

The evidence map is based on a systematic review of systematic reviews and summarizes healthcare research reporting on patient health outcomes on effects of Tai Chi. Systematic reviews provide comprehensive summaries of the literature for defined clinical topics by combining thorough and comprehensive searches and transparent synthesis of the available evidence. Systematic reviews often employ meta-analysis which provides the statistical power to identify small treatment effects by combining often small and underpowered studies.

We have registered this systematic review in PROSPERO (record number CRD42014009907). We report one deviation from the protocol: systematic reviews that do not include randomized controlled trials (RCTs) were not summarized in a narrative synthesis but were included in the bubble plot in the unclear category of the *x*-axis with the *y*-axis indicating that no RCT was identified despite an explicit search (see the “Data synthesis” section for more information). This manuscript is based on a comprehensive report for the Department of Veterans Affairs (VA), Veterans Health Administration, Office of Research and Development, Quality Enhancement Research Initiative, conducted within the Evidence-based Synthesis Program of the VA [3]. This manuscript aims to disseminate the finding to a broader audience of interested stakeholders. We report the methodology and results of the evidence map according to the PRISMA guidelines for systematic reviews to the extent possible (the PRISMA checklist is documented in Additional file 1). The VA report discusses the results within the context of the VA healthcare system and includes additional evidence synthesis results for VA-identified priority areas. This evidence map was supported by a technical expert panel of practitioner, policy maker, and researcher content experts.

### Data sources

We searched PubMed, CINAHL, Database of Abstracts of Reviews of Effects (DARE), Cochrane Database of Systematic Reviews (CDSR), Health Technology Assessments (HTA), Economic Evaluations (EED), Allied and Complementary Medicine (AMED), PsycINFO, Scopus, Web of Science, and PROSPERO from database inception to February 2014 for published English-language systematic reviews. In addition, we screened published reviews of reviews and consulted with topic experts. We used the terms “tai chi,” “tai-chi,” “tai ji,” “tai-ji,” “taiji,” “t’ai chi,” “t’ ai chi,” “taijiquan,” and “shadow boxing;” the full search strategy is documented in Additional file 2.

### Inclusion criteria

#### Design

Systematic reviews focusing on Tai Chi and summarizing primary research studies for all clinical indications were eligible for inclusion. We defined systematic reviews as reviews that either self-identified as a “systematic review” or reviews that reported the search sources and accounted for identified studies.

#### Participants

Systematic reviews of adult participants or unspecified age groups regardless of their health status were eligible for inclusion in the review; systematic reviews exclusively focusing on children and adolescents were excluded.

#### Intervention

Systematic reviews of the effects of Tai Chi for any clinical indication were eligible for inclusion. Systematic reviews addressing Tai Chi and other approaches were eligible if one of the two following criteria was met: (a) “Tai Chi” was part of the search strategy or (b) the search strategy did not specify any interventions *(*e.g., focused on an outcome) and the systematic review identified Tai Chi studies. We excluded systematic reviews that included Tai Chi studies but did not systematically search for these *(*e.g., by reviewing “exercise” interventions where only those Tai Chi studies were found that used the descriptive term “exercise”) and broad reviews on complementary and alternative medicine approaches without particular focus on Tai Chi.

#### Outcome

Systematic reviews reporting on patient health outcomes were eligible for inclusion. Systematic reviews of provider outcomes, acceptance, prevalence, use, costs, study design features, or intervention features not reporting patient health outcomes were excluded.

#### Timing

Systematic reviews summarizing evaluations of interventions of any duration and follow-up point were eligible for inclusion.

#### Setting

Systematic reviews of studies in healthcare-related settings were eligible for inclusion. English-language systematic reviews, regardless of the language of the included studies were eligible for inclusion.

### Procedure

**Fig. 1:**
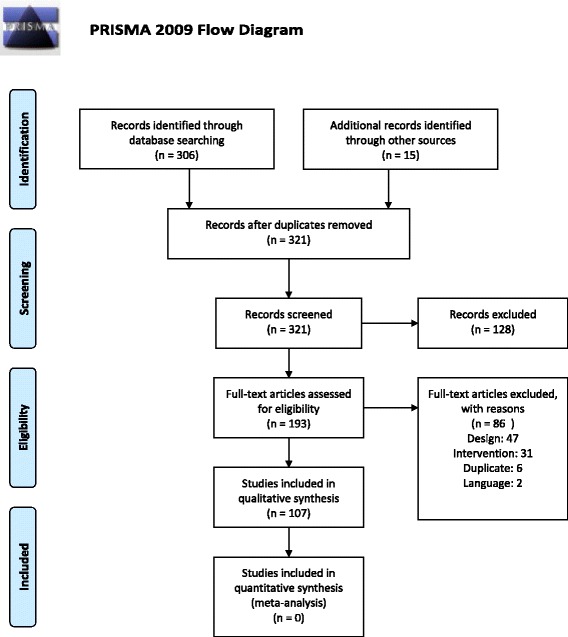
Literature flow

Two independent literature reviewers screened the systematic review search output. Citations deemed potentially relevant by at least one reviewer and unclear citations were obtained as full text. The full-text publications were screened against the specified inclusion criteria by two independent reviewers; disagreements were resolved through discussion. The reasons for exclusion of full-text publications were recorded (Fig. 1). Where originals and updates of systematic reviews by the same author group were available, only the most recent version was considered, and multiple publications of the same review were counted as one review but data were extracted from all available publications [5–8]. From each included systematic review, we extracted the specific clinical indication (e.g., osteoarthritis) and the main patient outcomes (e.g., balance) that were summarized across included studies. We extracted the number of Tai Chi RCTs included in the review, outcomes measured, comparators, treatment effect estimates for patient outcomes, and review characteristics. In addition, we documented which reviews were based on a format of Tai Chi that deviated from traditional formats (e.g., no weight shifting component; water-based; sitting, not standing; limited training intensity).

### Data synthesis

We used a bubble plot to visually display the Tai Chi research field.

#### Clinical indications (bubbles)

We used the topics of the identified systematic reviews to categorize the reviews. Reviews focused on outcomes, populations, or clinical indications. Systematic reviews groupings into clinical topics were drafted by one reviewer and discussed in the review team. Decisions not to combine potentially related topic areas (e.g., reviews on the outcome hypertension and reviews on patients with cardiovascular disease) were based on the lack of overlap between studies included in the reviews, differences in the reported outcomes, and differences in the review’s conclusion. All identified systematic reviews were allocated to a single content area and were only depicted once on the bubble plot.

#### Color

Indications that have been addressed in a publication by an agency specializing in unbiased evidence syntheses such as Cochrane and the Agency for Healthcare Research and Quality (AHRQ) are shown in dark green (all other bubbles are pale yellow).

#### Number of reviews (bubble size)

We used the size of the bubble to represent the number of systematic reviews on the topic

#### Literature size estimate (*y*-axis)

The bubble plot provides an overview of the research volume for each of the identified clinical indications. We used the number of RCTs per review, selecting the systematic review with the most included Tai Chi RCTs for the individual topic as the research volume estimate.

#### Effect estimate (*x*-axis)

The bubble plots provide a very broad indication of the clinical effectiveness of Tai Chi according to patient health outcomes reported in RCTs. All available systematic reviews were reviewed for each clinical indication noted on the evidence map. Greater significance was attributed to the largest review as it should provide the most complete literature synthesis, and reviews from agencies specializing in unbiased evidence syntheses as it should provide the most valid synthesis. Reviews reporting on only one RCT were classified as unclear evidence regardless of the statistical significance of the individual study given the paucity of the existing research and lack of replication of effect. For effect size estimates, meta-analytic results were sought to provide a summary effect across individual and often small and underpowered studies. Reviews reporting on studies with conflicting results across studies were classified as unclear evidence unless they reported a statistically significantly positive pooled effect estimate favoring Tai Chi or all included studies reported a positive effect of Tai Chi. The evidence map is divided into three sections: topics with evidence indicating potentially no effect (left section); topics for which the evidence base is unclear (middle section); and topics for which there is published evidence of a potential positive effect with a meta-analysis reporting statistically significant treatment effects of Tai Chi (right section).

## Results

**Fig. 2:**
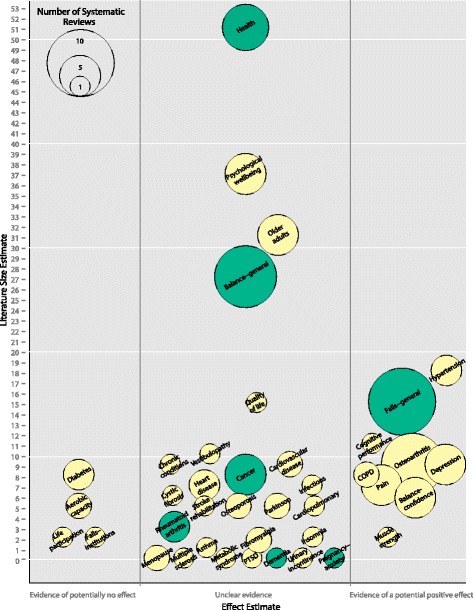
Evidence map of Tai Chi. The bubble plot displays Tai Chi research based on systematic reviews published to February 2014. *y*-axis: literature size estimate (number of RCTs included in the largest systematic review). *x*-axis: effect estimate (*three partitions*: evidence of potentially no effect, unclear evidence base, evidence of a potential positive effect). *Bubbles*: clinical indication. Color: *green bubbles* indicate that the identified systematic reviews on the topic include a Cochrane review or an AHRQ evidence report. Bubble size: number of systematic reviews on the topic

We identified 321 citations of which 107 unique systematic reviews met the criteria for inclusion in the review [5, 7, 9–113]. Figure 2 provides a graphic representation of the evidence base. Two thirds (66 %) of the reviews were published in the last five years and spanned a wide diversity of clinical indications, study populations, and outcomes.

### Size of research base

Topics with the largest research base included research on general health effects, psychological well-being, interventions in older adults, and effects on the outcome balance, hypertension, falls prevention, and cognitive performance.

The evidence base for the effectiveness of Tai Chi was unclear for the five of the largest areas of research. Six systematic reviews addressed positive effects of Tai Chi on health outcomes (Health) [7, 16, 36, 55, 59, 78]. The largest review, a comprehensive review of health benefits of qigong and Tai Chi, included 51 RCTs but did not provide treatment effect estimates across individual studies [55] and the other reviews primarily highlighted the need for more research. The reviews included studies that addressed a large range of outcomes. The largest review included studies that reported on 163 physiological and psychological health outcomes [55]. An AHRQ evidence report on meditation practices required studies to report measurable data for health-related outcomes and differentiated physiological (e.g., sensory outcomes), psychosocial (e.g., social and interpersonal relationships), and clinical outcomes (e.g., longevity) [7]. The other reviews differentiated the outcome categories balance improvement/postural stability/fall prevention, cardiovascular and ventilator enhancement, and other outcomes (rheumatoid arthritis, pain reduction, stress reduction, nightmare reduction); [16] included studies reporting on health outcomes such as cardio respiratory function, falls, balance, strength, or quality of life; [36] reported more than 22 different outcomes addressed in included studies and highlighted effects on quality of life, physical functioning, pain management, balance and risk of falls reduction, enhancing immune response, and improving flexibility/strength/kinesthetic sense; [59] or differentiated effects on cardiovascular disease, chronic disease and immunity, and psychological benefits [78].

Five systematic reviews concentrated on psychological well-being [24, 32, 56, 95, 111]. The largest review included 37 RCTs, but treatment estimates were only presented for three of the included RCTs [24]. A meta-analysis reported positive pooled results for a few selected outcomes, [95] two reviews did not provide specific treatment estimates, [56, 111] and one concluded that it is premature to form conclusions on the effect of Tai Chi on psychological well-being [32]. Four published systematic reviews have examined Tai Chi and qigong for older adults [84, 85, 94, 113]. The review authors focused specifically on this population and did not restrict the reviews to a particular clinical outcome. The reviews addressed a range of outcomes and analyses, including perceived benefits to health, perceived improved mediators such as social support, and perceived factors for initiating Tai Chi; [84] the efficacy of Tai Chi Chuan based on outcomes reported in included studies such as falls, balance, or cardiorespiratory functions; [94] physical and psychological health outcomes differentiating identified outcomes into the categories falls and balance, physical function, cardiovascular disease, and psychological and additional disease-specific responses; [85] and validated measures and self-reported indicators of mental well-being such as life satisfaction, mental health-related quality of life, self-esteem, or happiness and mastery [113]. The largest review included 31 RCTs; [85] none of the reviews reported specific treatment estimates for Tai Chi across studies.

The outcome balance has been addressed in nine systematic reviews by independent author groups [10, 37, 39, 53, 57, 61, 72, 77, 109] and the largest review included 27 RCTs. The largest review did not report treatment effect estimates [57] and an existing Cochrane review on exercise interventions included 12 Tai Chi RCTs but reported effects only for a combination of Tai Chi, gi gong, dance, and yoga interventions [53]. Another review pooled three RCTs and found no effect of Tai Chi on the single leg stance test compared to different control groups [10] while results of studies included in the remaining reviews varied and none of the reviews provided a treatment effect estimate across identified studies. A systematic review addressing health-related quality of life included 15 Tai Chi RCTs [28] but did not provide a summary estimate for Tai Chi effects and individual study results varied within and across studies.

### Potentially promising effects

Promising effects of Tai Chi, indicated by statistically significant pooled treatment effects in systematic reviews, and based on a substantial number of research studies included findings for hypertension, falls prevention outside of institutions, and cognitive performance. Hypertension has been addressed in three systematic reviews [68, 97, 106]. The pooled results of the largest review (18 RCTs) showed a larger number of participants with reduced blood pressure (relative risk [RR] 3.39; 95 % confidence interval [CI] 1.81, 6.34; 4 RCTs); reduced mean systolic blood pressure (mmHg WMD 12.43; 95 % CI 12.24, 12.62; 10 RCTs); and reduced mean diastolic blood pressure (mmHg WMD 6.03; 95 % CI 5.90, 6.16; 10 RCTs) compared to usual care [97]. However, the authors cautioned that the evidence remains weak and stated reservations due to the poor quality of the included studies, lack of longer follow-up, or conflicting results across outcomes, comparators, and settings. An earlier review that included only four RCTs in elderly participants concluded that the evidence for Tai Chi in reducing blood pressure in the elderly is limited, [68] and the third review did not provide a pooled treatment estimates across studies [106]. Tai Chi for fall prevention in unselected populations or participants living in the community (Falls-general) has been addressed in ten independent reviews [17, 20, 43, 47, 48, 51, 73, 75, 87, 101]. The largest review (15 RCTs) reported no benefit compared to non-exercise controls across five studies but found a significant pooled estimate for Tai Chi versus exercise controls (incidence rate ratio [IRR] 0.51; 95 % CI 0.38, 0.68; 2 RCTs); the review discussed a number of explanations for this finding, including a dose-response effect [73]. A Cochrane review on interventions for preventing falls in older people living in the community found no reduction in the rate of falls but reported a significantly reduced risk of falling (RR 0.71; 95 % CI 0.57, 0.87; 6 RCTs) associated with Tai Chi compared to diverse, predominantly passive comparators (e.g., wellness education) [47]. An AHRQ report on interventions to prevent falls in older adults included three Tai Chi RCTs, but no summary treatment effect was reported [20]. A further review reported a statistically significant pooled estimate for Tai Chi in community-dwelling participants (RR 0.66; 95 % CI 0.52, 0.78; comparators not specified) [17]. One review found no Tai Chi fall RCTs in older persons with cognitive impairment, and the remaining reviews did not provide a summary effect estimate. Of note, reviews in hospitals and nursing home settings (Falls-institutions) did not report positive findings [9, 17].

One systematic review on the effects of Tai Chi on cognitive performance in older adults identified 11 relevant RCTs [100]. This review found positive effects of Tai Chi on executive function in cognitively healthy adults compared to no intervention (SMD 0.90; *p* = 0.04; 4 RCTs) and exercise (SMD 0.51; *p* = 0.003; 2 RCTs), on global cognitive function in cognitively impaired adults compared with no intervention (SMD 0.35; *p* = 0.004; 4 RCTs) or other active interventions (SMD 0.30; *p* = 0.002; 4 RCTs). However, it cautioned that larger and methodologically sound trials with longer follow-up periods are needed before definitive conclusions can be drawn.

There are also a number of areas suggesting promising results but for which the volume of research is smaller and fewer than ten relevant RCTs were available to inform the reviews. Eight systematic reviews have addressed osteoarthritis [5, 12, 21, 30, 44, 63, 88, 90, 103], and the two largest reviews included nine RCTs each. One of them reported pooled results and showed positive effects of Tai Chi compared to different control groups on pain (SMD −0.79; 95 % CI −1.19, −0.39; 6 RCTs), physical function (SMD −0.86; 95 % CI −1.20, −0.52; 6 RCTs), and joint stiffness (SMD −0.53; 95 % CI −0.99, −0.08; 6 RCTs) but cautioned that due to the small number of RCTs with a low risk of bias, the evidence that Tai Chi is effective in patients with osteoarthritis is limited [5]. An independent review reported significant positive short-term effects for pain intensity (SMD −0.72; 95 % CI −1.00, −0.44; 5 RCTs), function (SMD −0.72; 95 % CI −1.01, −0.44; 5 RCTs), stiffness (SMD −0.59; 95 % CI −0.99, −0.19; 5 RCTs), and physical quality of life (SMD 0.88; 95 % CI 0.42, 1.34; 2 RCTs) but not for mental quality of life, or long-term effects for pain, physical function, and stiffness, compared to waitlist or attention control. The authors highlighted that all positive results represent short-term effects and high-quality RCTs are needed to confirm the results [63]. A 2013 meta-analysis reported statistically significant and clinically important effects for pain (SMD −0.45; 95 % CI −0.70, −0.20; 7 RCTs) across studies comparing Tai Chi to waiting list, Bingo, attention control programs, routine treatment, self-help programs, or wellness education and stretching, and concluded that 12-week Tai Chi programs should be included in rehabilitation programs but highlighted that the pain - relieving effect is not sustained and that additional studies are needed to investigate the long-term effects of Tai Chi in patients with knee osteoarthritis [103]. The remaining reviews did not identify eligible Tai Chi RCTs for their particular review question or did not report treatment effects across studies.

Positive outcomes were also reported in two reviews on chronic obstructive pulmonary disease (COPD) [42, 104] and the largest included eight RCTs. The largest review reported statistically significant pooled effects of Tai Chi for the 6-min walk test (WMD 34.22 m; 95 % CI 21.25, 47.20; 3 RCTs), dyspnea (WMD –0.86; 95 % CI –1.44, –0.28, 3 RCTs), forced expiratory volume in 1 s (WMD 0.07; 95 % CI 0.02, 0.13, 4 RCTs), forced vital capacity (WMD 0.12; 95 % CI 0.00, 0.23, 3 RCTs), and two quality of life measures (WMD 0.95; 95 % CI 0.22, 1.67; 2 RCTs; WMD −4.08; 95 % CI −7.52, −0.64; 3 RCTs), comparator not specified [104]. The second review combined Tai Chi and qigong interventions and did not provide treatment estimates across Tai Chi studies. Four systematic reviews have focused on the outcome pain [49, 81, 107, 112] and the largest review included seven RCTs (including six arthritis RCTs). The largest review found a positive effect of Tai Chi on self-reported pain (WMD 10.1 points on a 0–100 scale; 95 % CI 6.3, 13.9; 6 RCTs; comparators not specified) and self-reported disability (WMD −9.6; 95 % CI −14, −5.2; 4 RCTs) but not for physical performance, and data for quality of life were not pooled across studies [49]. Pooled treatment estimates of Tai Chi across studies were not reported in two other reviews, and one review found no eligible Tai Chi RCT. Five systematic reviews focused on balance confidence/fear of falling [15, 18, 33, 83, 91] and the largest included six RCTs. One reported a positive effect for Tai Chi compared to usual care, exercise, or education (SMD 0.47; 95 % CI 0.30, 0.63; 4 RCTs) [83]. The other reviews did not report a treatment effect estimate across studies. Five systematic reviews have specifically addressed the effects of Tai Chi on depression; [26, 34, 38, 93, 102] the largest review included four RCTs. It reported statistically significantly reduced depression symptoms (SMD −0.27; 95 % CI −0.52; −0.02; 4 RCTs) compared to waitlist in older adults but highlighted that further research is recommended with larger samples sizes, more clarity on trial design and the intervention, longer-term follow-up, and concomitant economic evaluations [38]. The other depression-specific reviews included only one or two Tai Chi studies or did not distinguish effects attributable to Tai Chi. However, the review of psychological well-being included nine RCTs reporting on depression, and it also reported a positive effect (Hedges’ *g* 0.48; 95 % CI 0.17, 0.78) [95]. A review on lower limb muscle strength in the elderly included two RCTs; both reported positive effects but did not report on the same outcome [11, 114].

### Evidence of no effect and unclear or conflicting evidence

The map includes a small number of systematic reviews that provide evidence of the potential lack of effectiveness of Tai Chi for clinical indications across more than one included study, e.g., fall prevention in hospitals and nursing homes (see the left hand side of the map). For these topics, systematic review authors concluded across identified studies that Tai Chi did not improve outcomes of interest; however, the number of existing studies in the identified topic areas was small in all of the identified topic areas.

In addition, unclear or conflicting evidence was found for a large number of topical areas as shown in the large middle section of the evidence map; in some cases, despite a number of existing systematic reviews that have attempted to synthesize the evidence in the research area.

### Lack of research

The evidence map also shows clinical topics that have been reviewed, but for which no Tai Chi studies could be found (*y*-axis = 0). Systematic reviews on menopause, dementia, metabolic syndrome, post-traumatic stress disorder (PTSD), urinary incontinence, multiple sclerosis, and anxiety during pregnancy systematically searched for Tai Chi studies. However, no RCTs, i.e., research studies supporting a high level of evidence, were identified in these systematic reviews.

### Other evidence base variables

Of the 107 included reviews, 42 % reported on the presence or absence of adverse events (not shown in Fig. 2). The large majority of these reviews noted that Tai Chi had little or no adverse effects on study participants. However, doing any exercise may put participants at greater risk and one review concluded that Tai Chi practiced by older adults may only be effective in a more robust older population and may not benefit frail participants [48]. None of the included reviews was exclusively based on Tai Chi interventions that deviated from traditional formats.

## Discussion

This evidence map for Tai Chi is based on 107 published systematic reviews and provides a broad overview of the available evidence of Tai Chi and its effect on patient outcomes. It shows the research concentration and the volume of available research and highlights areas where published meta-analyses have reported positive results.

Tai Chi has been evaluated for a wide range of clinical applications. Some identified systematic reviews included a large number of RCTs, but they addressed very broad topics such as health effects, psychological well-being, or interventions targeting older adults. On the other hand, evidence on the role of Tai Chi for a number of specific conditions is very limited due to the small number of published studies. Two thirds of identified systematic reviews included in this map were published very recently, i.e., in the last five years.

Although evidence maps can only provide a broad overview of research areas, it is noteworthy that across clinical topic areas, reviews concluded that more rigorous research on the clinical effectiveness of Tai Chi is needed. Furthermore, the effectiveness of Tai Chi may depend on several different factors including setting or patient characteristics—as indicated by differential effects of fall prevention in community versus hospitals or nursing homes. The optimal range of the Tai Chi intervention duration (short term versus long term) has not been determined, and a number of authors have indicated that more research on long-term effects is needed [63].

Our review of reviews also found that adverse events of Tai Chi have not been investigated systematically as noted in a recent review [115]. Given that the quality of the reporting of adverse events may depend on the standards in individual clinical fields, analyses across large, multi-indication reviews are particularly useful; a recent review concluded that much can be learned by comparing the effects of a given treatment across many related indications [114].

The evidence map—a visual overview of a systematic review of systematic reviews—is a new and unique review product that shows graphically, at a glance, the volume and focus of a research area through bubble color, size, and location. Based on a delineated systematic process (e.g., having specified search and inclusion criteria), evidence maps can be used to identify knowledge gaps and future research needs and to provide easily digestible and usable information from a large body of literature. Because evidence mapping is a relatively new and innovative evidence synthesis method, there are no established reporting guidelines; however, some principles have been articulated, including the use of an expert panel to ensure relevance and usefulness of the evidence, such as were used in this review [4].

The evidence map has several limitations. First, evidence maps cannot provide definitive answers about the effectiveness of an intervention. We used published reviews to provide an overview over the research on Tai Chi and did not undertake independent systematic reviews to calculate effect sizes in a meta-analysis, provide risk of bias assessments, or establish quality of evidence evaluations ourselves. Furthermore, the unit of analysis was systematic reviews, and individual primary research studies will have contributed to more than one included systematic review, in particular as reviews focused on different clinical indications, outcomes, or populations. In addition, the grouping of systematic reviews was review-content driven. The map did not follow a predefined structure and was unable to avoid overlap between included studies across reviews; the map was based on published reviews and used the topic structure of the reviews in order to explore the evidence base. The evidence map used review-level data, not primary research study data, and relied on the review authors’ clinical topic interest and skill in conducting systematic reviews. Furthermore, individual review conclusions may be limited by the quality of primary studies and susceptible to publication and outcome reporting bias.

Included Tai Chi interventions varied greatly by Tai Chi style, intervention duration, and intervention intensity; and studies varied in their choice of comparator to estimate the effectiveness of Tai Chi. A broad overview cannot answer more refined questions such as the effect of different styles of Tai Chi, the effect of the practitioner’s training, and skill level or the role of patients’ Tai Chi practice efforts. More specific results need targeted systematic reviews (addressing selected clinical indications and outcomes) and effect modifiers should be analyzed in meta-regressions designed to identify sources of heterogeneity across studies.

Future research should consider the body of evidence assembled in this map and systematically explore the effects of Tai Chi on clinically relevant outcomes across identified reviews. This broad overview has explored the research focus, as described in existing systematic reviews, and the map has identified several promising areas. Establishing more information on the effects of Tai Chi across and within clinical indications and patient populations, through meta-analyses across primary research studies, will further advance our evidence-based knowledge of Tai Chi. In addition, the large number of topic areas that were classified as unclear evidence warrants further research. Some topics addressed in reviews were very broad (“health,” “psychological well-being,” “older adults,” or “cancer”) and would benefit from targeted syntheses for specific outcomes. In other areas, there is a clear need for additional primary studies. The lack of positive or negative effect estimates is primarily a function of the absence of studies (Tai Chi effects on menopause, multiple sclerosis, metabolic syndrome, PTSD, dementia, urinary incontinence, asthma, and anxiety in pregnancy) at the time of the review. Finally, reviews for some of the topic areas included in the unclear evidence category have identified an emerging body of research, but summary effects estimating the treatment effect of Tai Chi are missing and should be addressed in future meta-analyses.

## Conclusions

Tai Chi has been applied in diverse clinical areas, and for a number of these, systematic reviews have indicated promising results. The evidence map provides a visual overview of Tai Chi research volume and content. Despite the outlined limitations, evidence maps provide valuable information on the landscape—the size, scope, and breadth—of a given domain of research. The visualization facilitates an easy and engaging overview and suggests evidence maps as a tool useful for a large array of stakeholders and for informing policy and clinical decision makers.

## Additional files

Additional file 1: PRISMA checklist. Additional file 2: Search strategy.
