# Supplementary material for: An evidence map of the effect of Tai Chi on health outcomes
Source: Syst Rev. 2016 Jul 27;5:126. doi: 10.1186/s13643-016-0300-y (PMC4962385; doi:10.1186/s13643-016-0300-y)
Supplement: Additional file 2: — Search strategy. [file 13643_2016_300_MOESM2_ESM.docx]

# Additional File

**TAI CHI SYSTEMATIC REVIEWS**

**SEARCH METHODOLOGY**

**DATABASE SEARCHED & TIME PERIOD COVERED:**

PubMed Clinical Queries Database – 1946-2/2014

**LANGUAGE:**

English

**SEARCH STRATEGY:**

"tai chi" OR tai-chi OR taiji OR "t'ai chi" OR "t' ai chi" OR taijiquan

AND

systematic[sb]

===========================================================================

**DATABASE SEARCHED & TIME PERIOD COVERED:**

PsycINFO – 1800’s-2/2014

**LANGUAGE:**

English

**SEARCH STRATEGY:**

"tai chi" OR tai-chi OR taiji OR "t'ai chi" OR "t' ai chi" OR taijiquan

AND

systematic OR meta-analy*OR metaanaly*

===========================================================================

**DATABASE SEARCHED & TIME PERIOD COVERED:**

Cochrane Database of Systematic Reviews – inception-2//2014

**LANGUAGE:**

English

**SEARCH STRATEGY:**

'"tai chi" OR tai-chi OR taiji OR "t'ai chi" OR "t' ai chi" OR taijiquan in Title, Abstract, Keywords

===========================================================================

**DATABASEs SEARCHED & TIME PERIOD COVERED:**

Cochrane Other Reviews (DARE), Health Technology Assessment (HTA), Economic Evaluations (NHS EED) – inception-2/2014

**LANGUAGE:**

English

**SEARCH STRATEGY:**

'"tai chi" OR tai-chi OR taiji OR "t'ai chi" OR "t' ai chi" OR taijiquan in Title, Abstract, Keywords

===========================================================================

**DATABASE SEARCHED & TIME PERIOD COVERED:**

AMED (Allied & Complementary Medicine Database) – 1985-2/2014

**LANGUAGE:**

English

**SEARCH STRATEGY:**

ab("tai chi" OR tai-chi OR taiji OR "t'ai chi" OR "t' ai chi" OR taijiquan) AND ab(systematic)

===========================================================================

**DATABASE SEARCHED & TIME PERIOD COVERED:**

CINAHL – 1981-2/2014

**LANGUAGE:**

English

**SEARCH STRATEGY:**

"tai chi" OR tai-chi OR taiji OR "t'ai chi" OR "t' ai chi" OR taijiquan

AND

systematic OR meta-analy*OR metaanaly*

===========================================================================

**DATABASE SEARCHED & TIME PERIOD COVERED:**

DARE via Centre for Reviews & Dissemination (CRD) – inception-2/2014

**SEARCH STRATEGY:**

"tai chi" OR tai-chi OR taiji OR "t'ai chi" OR "t' ai chi" OR taijiquan

===========================================================================

**DATABASE SEARCHED & TIME PERIOD COVERED:**

Scopus – inception-2/2014

**SEARCH STRATEGY:**

"tai chi" OR tai-chi OR taiji OR "t'ai chi" OR "t' ai chi" OR taijiquan

AND

systematic OR meta-analy*OR metaanaly*

===========================================================================

**DATABASE SEARCHED & TIME PERIOD COVERED:**

Web of Science – inception-2/2014

**SEARCH STRATEGY:**

"tai chi" OR tai-chi OR taiji OR "t'ai chi" OR "t' ai chi" OR taijiquan

AND

systematic OR meta-analy*OR metaanaly*

===========================================================================

**DATABASE SEARCHED & TIME PERIOD COVERED:**

PROSPERO – inception-5/2014

**SEARCH STRATEGY:**

title field (tai chi OR tai-chi OR taiji OR t'ai chi OR taijiquan)
